# Artificial Intelligence in Prehospital Emergency Care: Advancing Triage and Destination Decisions for Time-Critical Conditions

**DOI:** 10.7759/cureus.91542

**Published:** 2025-09-03

**Authors:** Rayan Zarei, Martin C Downs, Leslie Torgerson

**Affiliations:** 1 Department of Biomedical Sciences, Rocky Vista University College of Osteopathic Medicine, Parker, USA

**Keywords:** acute coronary syndrome, artificial intelligence, emergency medical services, machine learning, prehospital triage, respiratory distress, sepsis, stroke, time-critical conditions, trauma

## Abstract

Prehospital triage is critical for time-sensitive emergencies such as trauma, stroke, and acute coronary syndrome. Undertriage delays definitive care, while overtriage strains higher-level facilities. Existing triage tools based on vital signs and scoring systems have limited accuracy, but artificial intelligence (AI), machine learning (ML), and neural networks (NN) offer the potential to improve decision-making by integrating multiple data sources. This narrative review of studies indexed in PubMed and PubMed Central through August 2025 evaluated AI, ML, and NN models designed for prehospital triage or transport destination decisions across trauma, critical illness, stroke, dyspnea, cardiac emergencies, sepsis, and in-hospital studies preventing possible readmission. Across the conditions examined, ML models consistently outperformed traditional early warning scores and guideline-based tools. Trauma models achieved area under the curve (AUC) values between 0.75 and 0.93 and reduced undertriage to less than 10%. ML models predicted the need for critical care with an AUC of 0.908, and prehospital stroke algorithms reached AUCs above 0.98. NN, deep forest, and random forest models demonstrated an AUC of 0.88 in prehospital acute respiratory distress syndrome (ARDS) prediction. Additional studies demonstrated improved recognition of dyspnea-related serious events and acute coronary syndrome, while no validated models currently exist for prehospital sepsis. Despite promising results, most studies were retrospective, with limited prospective validation, generalizability, or evaluation of workflow integration. Future research should focus on prospective studies, diverse patient cohorts, integration into emergency medical service (EMS) workflows, model explainability, and rigorous comparisons with standard practice.

## Introduction and background

Time-sensitive emergencies such as major trauma, stroke, myocardial infarction, and sepsis require rapid recognition and transport to the most appropriate facility. Emergency medical services (EMS) clinicians often must decide within minutes whether to take a patient to a comprehensive trauma or stroke center, a local hospital, or a specialized service [1]. Current prehospital triage relies on physiological thresholds and simple scoring systems. While widely adopted, these methods have limited sensitivity and may miss patients with severe illness, leading to undertriage. Conversely, they can also generate high false-positive rates, contributing to overtriage and unnecessary use of advanced resources [2]. In the United States, undertriage rates exceeding 30% for severe trauma have been reported, placing patients at risk of delayed care. Additional challenges include high cognitive load, time pressure, and variable provider training, all of which can further reduce accuracy.

Artificial intelligence (AI), machine learning (ML), and neural networks (NN) offer an opportunity to strengthen prehospital decision-making. By analyzing large and complex datasets, these models can identify patterns not easily recognized by clinicians. They are capable of integrating multiple features, including vital signs, mechanism of injury, dispatch information, and contextual factors, to generate probabilistic predictions of severe injury or critical illness. Reviews of AI in emergency medicine highlight that decision points extend from dispatch through hospital disposition, yet most existing work has focused on in-hospital applications.

To the best of our knowledge, to date, no comprehensive literature review has specifically examined AI-based triage models in the prehospital setting across multiple time-critical conditions. This review addresses that gap by evaluating current evidence on AI, ML, and NN for prehospital triage and destination decisions and by outlining key opportunities and challenges for future research.

## Review

Methods

We conducted a narrative review of studies indexed in PubMed and PubMed Central up to 14 August 2025. Search terms included combinations of “artificial intelligence,” “machine learning,” “deep learning,” “prehospital,” “triage,” “emergency medical services,” “paramedic,” “ambulance,” “destination decision,” and condition-specific keywords (e.g., “trauma,” “stroke,” “myocardial infarction,” “dyspnea,” “sepsis,” “aortic syndrome”). Reference lists of relevant articles were also screened to capture additional studies.

**Figure 1:**
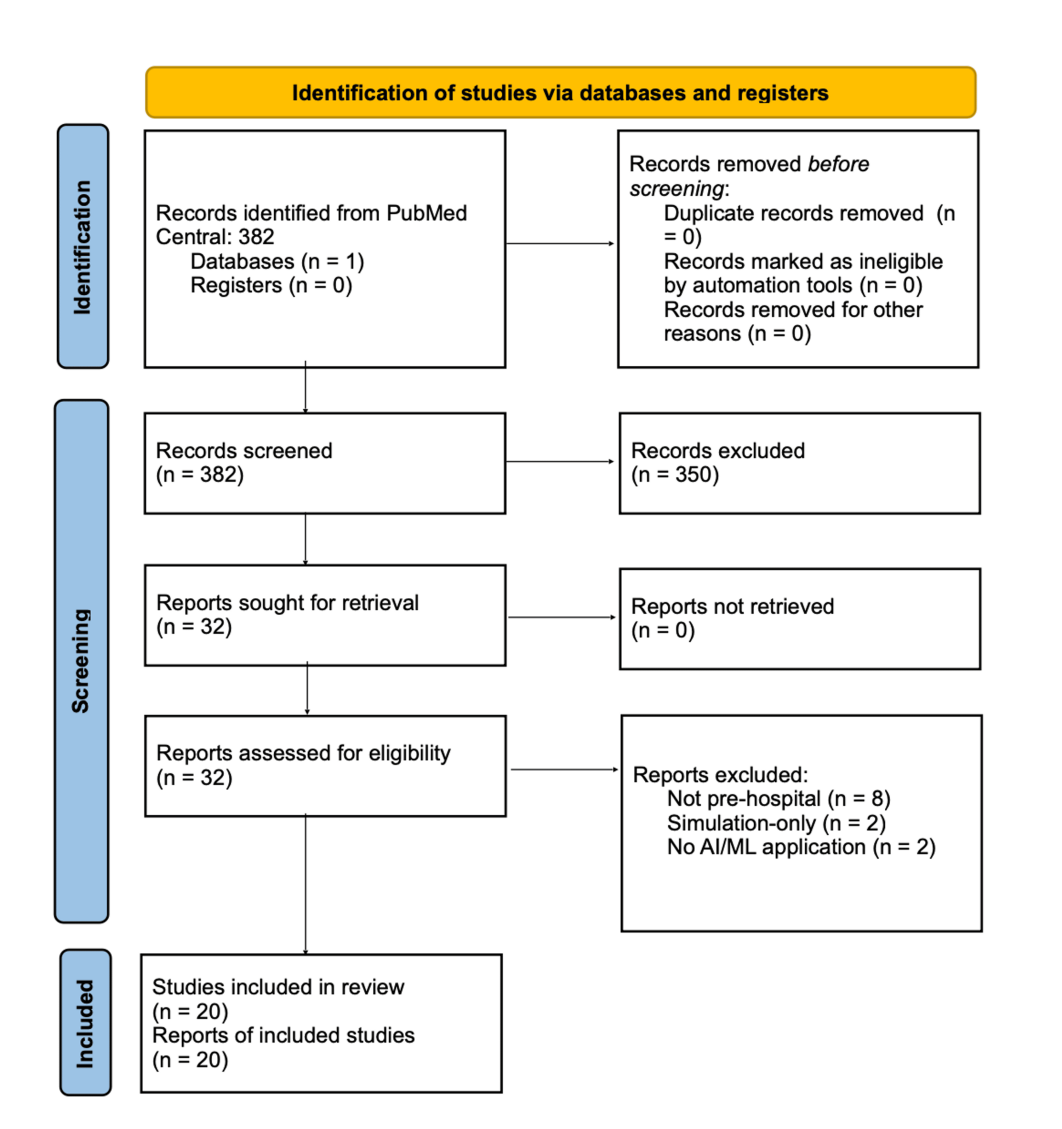
PRISMA-style flow diagram of study selection PRISMA: Preferred Reporting Items for Systematic Reviews and Meta-Analyses; AI: artificial intelligence; ML: machine learning

The initial search yielded 382 articles. After removal of duplicates and title/abstract screening, 350 articles were excluded for irrelevance (e.g., in-hospital only, case reports, or non-AI studies). Full-text review excluded an additional 12 studies that did not meet the inclusion criteria. Ultimately, 20 articles were included in this review. The study selection process is outlined in Figure 1.

We included original research articles, both prospective and retrospective, that developed or evaluated AI/ML models to support prehospital triage decisions, early identification of serious conditions, or transport destination planning. Studies applying ML to dispatch performance, early warning scores, or destination decisions were eligible. Reviews, commentaries, and qualitative studies were considered if they provided insights into prehospital AI applications or barriers to adoption. Case reports, single-patient simulations, and purely in-hospital triage studies were excluded. In-hospital studies were included only if they had relevance to reducing readmissions or preventing future emergency department (ED) overcrowding.

For each eligible study, we extracted details on study design, population, data sources, features, algorithm type, outcomes, and performance metrics. When available, discrimination statistics (e.g., area under the receiver operating characteristic curve (AUROC)), calibration measures, sensitivity, specificity, predictive values, Brier score, F1 score, and under- or overtriage rates were recorded. Owing to heterogeneity in study design and outcomes, a formal meta-analysis was not feasible. Instead, findings were synthesized qualitatively and organized by condition (trauma, critical care, stroke, dyspnea, cardiac emergencies, and sepsis/aortic disorders).

Trauma and severe injury

Several studies have evaluated AI models to improve the identification of patients with severe trauma who require transport to level I or II trauma centers. Chen and colleagues developed a field triage model (pTEST) using extreme gradient boosting trained on more than two million records from the National Trauma Data Bank [2]. Candidate predictors included age, mechanism, and patterns of injury, vital signs, and prehospital interventions. In internal and external validation sets, pTEST achieved an area under the curve (AUC) of 0.755 for predicting Injury Severity Score ≥ 16 and 0.736 for predicting the need for critical resources, outperforming the Glasgow Coma Scale, Revised Trauma Score, and National Field Triage Guidelines [2]. At a specificity of 0.5, the sensitivity for severe trauma was 0.799, and the undertriage rate was 8%. Undertriage was reduced by approximately 50% relative to conventional tools, although overtriage remained high.

Weidman et al. created an ML trauma triage model for critical care transport using physiological waveform data from helicopter transports [3]. Gradient boosting, random forests, and logistic regression were compared. The best model achieved an AUROC of 0.810 for predicting the need for lifesaving interventions, with specificity of 0.960 and sensitivity of 0.268 [3]. Sensitivity increased to 0.787 when overtriage rates were matched to standard guidelines, suggesting the potential to streamline critical care transport decisions.

Kim et al. developed a data-driven AI model for remote triage in mass casualty settings using 460,444 trauma cases from the National Trauma Data Bank [4]. NN using prehospital vital signs and a simplified consciousness score achieved an AUC of 0.89, outperforming the Revised Trauma Score (AUC 0.78); inclusion of the Injury Severity Score increased performance to an AUC of 0.93 [4]. The authors suggested that the model could be embedded in wearable devices to automate triage during disasters.

Prediction of critical care need

The challenge of identifying patients who will require intensive care or lifesaving interventions begins at the time of EMS contact. Kang and colleagues developed a deep learning algorithm to predict the need for critical care based on age, sex, chief complaint, symptom onset to arrival time, trauma mechanism, and initial vital signs [5]. Trained on 8.98 million ED visits and validated on 2,604 prehospital run sheets, the model achieved an AUROC of 0.867 and outperformed the Emergency Severity Index (AUROC 0.839), Korean Triage and Acuity System (0.824), National Early Warning Score (0.741), and Modified Early Warning Score (0.696) [5]. The authors argued that such models could assist EMS providers in determining whether patients require transport to centers with intensive care capabilities. A retrospective study with over 2,937,078 pediatric patients across 151 EDs in South Korea showed that deep learning modules significantly outperformed traditional pediatric predictive models for the prediction of the need for critical care hospitalization. Deep learning had an AUC of 0.980. On the other hand, the traditional models tested included the Pediatric Early Warning Score, which had an AUC of 0.812, and the Conventional Triage and Acuity System, which had an AUC of 0.782 [6].

Stroke

Early recognition of stroke subtype and severity is critical because patients with large vessel occlusion (LVO) benefit from mechanical thrombectomy at comprehensive stroke centers. Hayashi et al. prospectively collected prehospital symptom and vital sign data and trained gradient boosting, random forest, support vector machine, and logistic regression models [7]. XGBoost achieved AUCs of 0.994 during training and 0.980 in the test cohort for distinguishing stroke from nonstroke conditions [7]. Important features included sudden headache, unilateral paralysis, convulsions, impaired consciousness, elevated systolic blood pressure, and arrhythmia [7]. The models also differentiated between stroke subtypes; in the test cohort, the AUROC for predicting ischemic stroke with LVO was 0.898 and for subarachnoid hemorrhage 0.926 [7]. Such algorithms could support EMS crews in directing patients to the appropriate stroke center.

Prehospital management also starts with preventing returns to the hospital in the first place; AI can assist here as well. A randomized controlled trial from 2023 was the first to use deep learning with diffusion-weighted MRI (DWI), combined with using the modified Rankin Scale (mRS) in predicting 90-day outcomes of acute ischemic stroke (AIS) patients, rather than just using binary outcomes. Through the use of imaging and clinical data, results showed that the internal cohort (deep learning model trained on) showed an AUC of 0.92 for the prediction of unfavorable outcome, while the external cohort (deep learning model not trained on) showed an AUC of 0.90 for unfavorable outcome [8]. The ability of this model to integrate different types of unique medical data shows great promise for the future of stroke outcomes and preventing overflow of triage long before EMS is called.

Additional work has explored AI-assisted detection of stroke signs and dispatch decision-making. Computer vision algorithms integrated into smartphones or wearable devices have reported 97% accuracy in detecting facial droop and 72% accuracy in recognizing arm weakness [9]. Automated speech recognition algorithms used in dispatch centers increased stroke detection by 16% and thrombolysis utilization by 2% [9]. AI-enabled telemedicine platforms combining video examination and eyetracking have predicted LVO with a sensitivity of 0.70 and a specificity of 0.93 [9]. Novel diagnostic tools, such as EEG caps and transcranial Doppler devices paired with AI classifiers, have discriminated LVO strokes with accuracies around 91%, although these have not yet been validated in real prehospital environments [9]. Qualitative research involving EMS providers suggests that paramedics view AI as an adjunct that must be trustworthy, portable, and able to distinguish between hemorrhagic and ischemic strokes [10].

Traditional early warning scores

Ward et al. compared gradient boosting, random forests, logistic regression, and Bayesian networks with NEWS2 and the Danish Emergency Process Triage using 219,323 ambulance records [11]. ML models showed higher AUROC and area under the precision-recall curve across all thresholds [11]. When sensitivity was matched, the gradient boosting and Bayesian network models reduced false positives compared with NEWS2 and DEPT, indicating that ML could reduce overtriage without compromising sensitivity [11].

Dyspnea and respiratory distress

Kauppi et al. developed an ML-based decision support tool for prehospital assessment of dyspnea using data from 6,354 ambulance patients [12]. Gradient boosting outperformed logistic regression, least absolute shrinkage and selection operator, and existing triage tools (Rapid Emergency Triage and Treatment System - Adult (RETTS-A) and National Early Warning Score 2) for predicting serious adverse events such as death within 30 days, time-sensitive diagnoses, or intensive care admission [12]. The ROCAUC improved from 0.73 with RETTS-A to 0.81 with gradient boosting, and sensitivity increased, suggesting that ML models could better identify high-risk dyspnea patients [12].

Furthermore, an observational cohort study successfully demonstrated that AI can be used to prevent acute respiratory distress syndrome (ARDS). This study was conducted at two different Chinese hospitals involving patients who underwent cardiac surgery, and used 13 perioperative predictors in the prediction of ARDS development. Deep and random forest models were used, yielding AUC and Brier scores of 0.882 and 0.809, significantly outperforming traditional clinical scoring models. This study overall demonstrates that AI can be used to mitigate ARDS risk in patients; however, this is in a limited population. A wider scope of patients and data should be pursued in the future to validate the use of AI in this pathology [13].

Lastly, asthma exacerbations in pediatric patients are unfortunately often a cause of a trip to the ED. A retrospective observational study demonstrated that a multi-layer perceptron NN model outperformed traditional rule-based models in the detection of these exacerbations. The NN specifically yielded a sensitivity of 0.91, a specificity of 1.00, a negative predictive value (NPV) of 0.98, a positive predictive value (PPV) of 1.00, and an F1 score of 0.95 [14].

Acute coronary syndrome and cardiac emergencies

Takeda and colleagues assessed ML algorithms for early diagnosis of acute coronary syndrome using 43 prehospital features, including symptoms, three-lead electrocardiography, and vital signs [15]. The random forest-based voting classifier achieved a test AUC of 0.861, and performance was maintained when features were reduced to 17 variables (AUC 0.864) [15]. In an external validation cohort, XGBoost predicted acute coronary syndrome with an AUC of 0.840 [15]. These models outperformed logistic regression and support vector machines and indicate that limited-feature models can be implemented feasibly by EMS personnel to determine whether patients should be transported directly to cardiac centers. A recent retrospective study from the Netherlands also trained an NN model using EMS data to predict acute coronary syndrome before hospital arrival. Although detailed metrics were not published, the study demonstrates growing interest in AI-assisted cardiac triage.

An observational study from 2024 showed that AI-driven quantitative analysis of coronary plaque features and hemodynamics significantly increased the accurate prediction of ACS. Through the analysis of 351 patients who had undergone computed tomography angiography (CTA) one month to three years before an ACS event, AI increased the AUC from 0.78 to 0.84 compared to the reference model. A total of 363 culprit lesions and 2,088 nonculprit lesions were identified in patients through AI [16].

In evaluating myocardial infarctions, the use of troponin I is one of the most common diagnostic methods. Toprak and colleagues demonstrated that AI-ARTEMIS, combined with a high-sensitivity point-of-care (POC) troponin I, can rule out myocardial infarctions twice as fast and in twice as many patients compared to standard procedures [17]. Although this isn’t strictly a pre-hospital setting, this demonstrates that AI can correctly analyze and process high-sensitivity lab tests in patients. Ultimately, this could improve the ED flow of patients in the future.

Sepsis and other conditions

Despite the importance of early sepsis recognition, no studies have yet developed or validated ML models using purely prehospital data for sepsis. An integrative review of ML approaches for sepsis identification noted that existing models based on ED data outperform traditional statistical models but emphasized the absence of prehospital data and called for future integration of ambulance vital signs and patient history [18]. A cohort study geared towards predicting the onset of sepsis in ICU patients demonstrated that an AI random forest algorithm had a specificity of 89%, sensitivity of 87%, and AUC of 0.91 in the prediction of sepsis. Over 55 clinical variables for each patient were used, with a cohort of 4,449 patients [19]. Though this study was not strictly involving the pre-hospital setting, the AI performance in detecting sepsis in ICU patients was deemed highly successful.

Similarly, acute aortic syndrome is a rare but time-sensitive condition that can mimic other thoracic emergencies. One study used logistic regression and an ensemble SuperLearner to predict acute aortic syndrome prehospital; while results suggested improved discrimination compared with physician assessment, the lack of accessible full text precluded extraction of quantitative metrics [20]. These gaps highlight opportunities for AI research beyond trauma, stroke, and cardiac emergencies. A summary of representative studies, model types, datasets, and reported performance is provided in Table 1 [2-20].

**Table 1:** Summary of artificial intelligence and machine learning models for prehospital triage across time-critical conditions AUC: area under the curve; LVO: large vessel occlusion; EMS: emergency medical services; ED: emergency department; ARDS: acute respiratory distress syndrome; AIS: acute ischemic stroke; MI: myocardial infarction; ICU: intensive care unit; AI-QCPHA: artificial intelligence-enabled quantitative coronary plaque and hemodynamic analysis; NN: neural network

Condition	Representative Studies	Model Type	Dataset/Source	Best Reported Performance	Key Limitations
Trauma	Chen et al. [2], Weidman et al. [3], Kim et al. [4]	Gradient boosting, neural networks, and random forests	National Trauma Data Bank (>2M cases), helicopter transport data	AUC 0.75-0.93; undertriage <10%	Mostly retrospective; high overtriage persists
Critical Care Prediction	Kang et al. [5], Kwon et al. [6]	Deep learning, machine learning	8.9M ED visits, validated on 2,604 EMS runs, 2.9M ED visits across 151 EDs, 264,976 EMS calls	AUC 0.908 for critical care (outperformed pediatric early warning score, conventional triage and acuity system, random forest, and logistic regression)	Limited external validation; ED-heavy dataset, retrospective study
Stroke	Hayashi et al. [7], Wolcott et al. [9], Liu et al. [8]	Gradient boosting, random forests, CV/telemedicine AI, XGBoost	Prospective EMS cohort; smartphone/telemedicine tools, only AIS patients.	AUC 0.98 for stroke ID; 91% accuracy for LVO (experimental)	Not yet validated in real-world EMS workflows, retrospective, and limited cohort size
Dyspnea/Respiratory Distress	Kauppi et al. [12], Zhang et al. [13], Harmon et al. [14]	Gradient boosting, deep forest, Random forest, multi-layer perceptron NN	6,354 ambulance patients, 24,283 eligible patient encounters	AUC 0.88 in ARDS prediction	Lacks external testing and validation
Cardiac (ACS)	Takeda et al. [15], Koo et al. [16], Toprak et al. [17]	Random forest, XGBoost, AI-QCPHA, ARTEMIS	EMS features (symptoms, 3-lead ECG, vitals), MI symptoms (chest pain)	AUC 0.84-0.86	Limited feature sets; retrospective
Sepsis	Desai et al. [18], Wang et al. [19]	Random forest	ED-based and ICU models only	AUC 0.91 in ICU sepsis prediction	Research gap - no prehospital-specific data, retrospective, limited input variables
Aortic Syndrome/Rare Conditions	Duceau et al. [20]	Logistic regression, ensemble	Small prehospital datasets	Improved vs. clinician gestalt	Sparse data; limited reporting

Discussion

AI, ML, and NN demonstrate substantial promise in improving prehospital triage accuracy and destination decisions across multiple emergency conditions. Across trauma, stroke, cardiac emergencies, and respiratory distress, ML-based models consistently outperformed guideline-based scores and early warning systems. In trauma, gradient boosting and NN reduced undertriage to below 10% while maintaining or reducing overtriage [2,4]. Physiological waveform data and simplified consciousness scales captured information beyond static vital signs and improved prediction of the need for lifesaving interventions [3,4]. For stroke, AI algorithms accurately distinguished ischemic subtypes, predicted LVO, and improved dispatcher recognition and outcome prediction [7-9]. Cardiac, ARDS, and dyspnea models achieved AUCs in the range of 0.80-0.88, outperforming common prehospital triage tools [12-15]. Functional outcome models in AIS also reached AUCs around 0.90-0.92, suggesting strong potential for integration into clinical workflows [8].

Despite these encouraging results, several limitations temper enthusiasm. Many studies used retrospective datasets and lacked prospective validation, which may overestimate performance due to overfitting or selection bias. Even when external validation was performed, geographic and demographic diversity was limited, raising concerns about generalizability to low-resource settings. Because of the heterogeneity of study designs and restriction to English-language publications, selection bias is possible, and relevant studies may have been missed. Model interpretability also varied, and “black box” algorithms may be less acceptable to clinicians and regulators. Qualitative studies emphasize that EMS providers view AI as an adjunct rather than a replacement, requiring transparency, trust, and the ability to override algorithmic recommendations [10].

Beyond predictive performance, deployment raises ethical and medicolegal challenges. Algorithmic bias is a significant concern, as models trained on homogenous datasets may underperform in underrepresented populations, potentially exacerbating disparities. Safeguarding sensitive health data in mobile and wireless prehospital environments also poses risks. Medicolegal accountability remains unresolved: if an AI recommendation conflicts with EMS judgment and results in harm, responsibility is unclear. Regulatory oversight will be necessary to ensure that models meet safety, transparency, and accountability standards before widespread adoption.

The literature also highlights gaps in conditions beyond trauma, stroke, and acute coronary syndrome. For sepsis, no validated prehospital-specific ML models currently exist despite strong evidence that early recognition and expedited antibiotic delivery reduce mortality. While ICU- and ED-based models show promising performance, the lack of integration into EMS data streams prevents truly early intervention [18,19]. Similarly, acute aortic syndrome is a rare but lethal condition frequently misclassified as acute coronary syndrome or pulmonary embolism in the prehospital setting. AI-assisted triage capable of distinguishing these emergencies could reduce diagnostic delays and expedite definitive care. Expanding research in these areas would address critical unmet needs.

To provide tangible clinical benefit, AI must integrate seamlessly into EMS workflows. Algorithms embedded into ambulance monitors, tablets, or dispatch systems could support risk stratification without disrupting operations. Practical tools must deliver interpretable outputs quickly, aligning with existing triage protocols rather than replacing them, to preserve EMS clinician autonomy. Training programs and user-friendly interfaces will be essential to encourage adoption, and explainable AI techniques, such as Shapley additive explanations, may enhance clinician trust by clarifying how predictions are generated. Integration must occur in a way that augments, rather than slows, time-critical decision-making.

Future research should prioritize prospective, multicenter studies to validate these models in diverse populations and settings. Federated learning approaches could facilitate collaboration across EMS systems without compromising patient privacy. Beyond accuracy, downstream outcomes such as mortality, neurological recovery, and healthcare costs must be evaluated to determine whether AI-enhanced triage improves patient care. Attention to ethical and legal challenges, mitigation of bias, and interoperability with EMS systems will be central to safe implementation. With thoughtful design and rigorous validation, AI has the potential to transform prehospital care by ensuring patients with time-sensitive emergencies receive the right care at the right place and time.

Limitations

Several limitations in this study are important to mention. First, the literature search was restricted to PubMed and PubMed Central, which may have excluded relevant studies indexed in other databases. Because the included studies were heterogeneous in design, population, and outcome measures, a meta-analysis could not be performed, and findings were synthesized qualitatively. Most of the available evidence is retrospective, derived from limited geographic regions, and focused primarily on trauma, stroke, and cardiac emergencies, leaving conditions such as sepsis, pulmonary embolism, and aortic syndromes underexplored. Additionally, few studies evaluated real-world deployment, user acceptability, or downstream patient outcomes. These limitations should be considered when interpreting the findings and underscore the need for prospective, multicenter trials to establish the true clinical impact of AI-enabled prehospital triage.

## Conclusions

AI and ML have the potential to transform prehospital emergency care by improving triage accuracy and destination decisions. Current evidence suggests that these models can outperform conventional scoring systems and reduce both undertriage and overtriage in conditions such as trauma, stroke, cardiac emergencies, and respiratory distress, with algorithms demonstrating high accuracy in predicting severe trauma, LVO, and the need for critical care. However, most studies remain retrospective, limited to a small number of conditions, and based on narrow populations. There is little evidence regarding real-world deployment, user acceptability, ethical considerations, or patient-centered outcomes. To fully realize the benefits of AI in prehospital medicine, future work must include rigorous prospective trials, evaluation across diverse populations, attention to fairness and transparency, and close collaboration with EMS providers to ensure that these tools complement rather than replace clinical judgment. With careful integration, AI could enhance the speed and precision of triage and ensure that patients with time-sensitive emergencies receive the right care at the right place and time.
